# Supplementary material for: Surveillance of invasive Aedes mosquitoes along Swiss traffic axes reveals different dispersal modes for Aedes albopictus and Ae. japonicus
Source: PLoS Negl Trop Dis. 2020 Sep 28;14(9):e0008705. doi: 10.1371/journal.pntd.0008705 (PMC7544034; doi:10.1371/journal.pntd.0008705)
Supplement: Attachment — Submitted filename: renamed_47604.docx [file pntd.0008705.s003.docx]

PLOS
Carlyle House
Carlyle Road
Cambridge, CB4 3DN
United Kingdom

Basel, 2 August 2020

**Re-submission PNTD-D-20-00715**

Dear Editors,

Many thanks for sending the positive feedback and helpful reviews for our earlier submission of the manuscript entitled “Surveillance of invasive *Aedes* mosquitoes along Swiss traffic axes reveals different dispersal modes for *Aedes albopictus* and *Ae. japonicus*”.

We have taken into account the points raised by the reviewers and provided a point-by-point response below. We hope the points raised have been adequately addressed for the paper to be published in PLOS NTD.

We look forward to reading from you in due course.

Sincerely yours,

Dr Pie Müller (on behalf of the authors)

*Head Vector Control Group*

*Department of Epidemiology and Public Health*

**Point-by-point response**

Reviewer #1: The authors monitored alien Aedes mosquitoes along Swiss traffic axes with ovitraps (egg collection) and BG Sentinel traps (adults) – from June-September 2013-2018. Aedes albopictus, Ae. japonicus and Ae. koreicus were documented. Based on their results Ae. albopictus and Ae. koreicus are passively spread while Ae. japonicus is spreading actively.

This manuscript is of excellent quality and provides important information about the alien Aedes mosquito situation in Switzerland. It is also of high relevance for public health.

Some minor modifications are recommended (and I apologize that I mention our studies but it would be of interest to compare and discuss)

Line 20: You could also include Zika here

*Authrors' response: We have added "Zika virus" on line 20.*

Line 38: These Aedes mosquitoes are mainly of relevance for human medicine

*Authors's response: We swapped "human" and "veterinary" to emphasise its primary role in public health.*

entire manuscript: Please unify – Ae. japonicus or Ae. japonicus japonicas

*Authors's response: While we still refer to* "Ae. japonicus japonicus", *when first mentioned we have now emphasized that it is referred to as* "Aedes japonicus" in the remaining text*. See line 96.*

entire manuscript: use of invasive – We all know that Ae. albopictus is an invasive species - however, the status of invasiveness in the Central and Eastern Alpine regions remain unclear (outcompetition of indigenous mosquitoes beside establishment). Therefore, it is recommended to use potential invasive or alien

Authors' response: *Although we agree that it remains to be shown to what extent* Ae. albopictus *may establish stable populations across the Alpine region, we would still prefer keeping the term "invasive" throughout the article because it refers to the species and Switzerland as a whole. The question about potential "invasiveness" has been addressed in the Discussion section (see lines 465 – 447).*

Fuehrer et al. (2020 – PLoS NTDs) published a study about these mosquitoes at high ways in the Eastern Alps region of Austria (Tyrol) a few days ago. It is recommended to compare results.

*Authors' response: Thanks for pointing this out as this article has not yet been published at the time of submitting our manuscript. We have added the several references to this article:*

*Lines 129 - 131: "Today,* Ae. koreicus *is present in at least seven European countries, including Austria, Belgium, Germany, Hungary, Italy, Slovenia and Switzerland (30, 40-42, 44-46)."*

*Lines 371 - 375: "Likewise, the passive spread of* Ae. albopictus *from south to north would explain our repeated findings along the San Bernardino route A13, an alternative south-north passage way through Switzerland. A similar picture emerges from a study in western Austria (Tyrol) where service stations along the motorways coming from Italy were repeatedly positive (44)."*

*Lines 416 – 417: "Moreover,* Ae. koreicus *has also been repeatedly reported from Germany (30, 62, 63) and more recently from Austria (44)."*

Line 118: Please provide information which BG-Sentinel generation was used

Authors' response: *We have changed "BG Sentinel" to "BG Sentinel version 1" to indicate the generation of traps deployed (see line 139).*

Line 228: New paragraph after koreicus

Authors' response: *We feel the paragraph should not be split because the sentences belong to the same topic. However, we moved the sentence in line 228 to the end of the paragraph for a better flow of the text (see line 259 - 260).*

Change Culex pipiens s.s. to Culex pipiens complex

Authors' response: *These specimens were identified as* Culex pipiens *s.s. based on validated MALDI-TOF MS spectra and we can exclude* Culex quinquefasciatus (other complex member) *or* Culex torrentium (other sibling species). *While we cannot differentiate between the two biotypes (i.e.* Culex pipiens pipiens *and* Cx. pipiens molestus*)* *both of them would - to the best of our knowledge - still be considered* as Culex pipiens *s.s. Therefore, we feel it is appropriate to keep the species name as is.*

General question: Did you observe any altitude differences? We observed Ae. japonicus in mountainous areas above 850 m (Schoener et al. 2019)

Authors' response: Many thanks for raising this question. *We found both* Ae. albopictus *and* Ae. japonicus *at the highest site at 1,064 m (i.e. A2 San Gottardo-Sud Stalvedro) as well as at the lowest site 227 m (i.e. A2 Bellinzona-Sud). This is an interesting fact and we have now highlighted this in the Results section (see line 306 - 308).*

Line 383: Change , to .

Authors' response: *Done.*

Line 407: Cx. torrentium abundance – change to “… north of the Swiss Alps” or modify – The statement is not true for the entire northern to the Alps regions (e.g. Zittra et al. 2016; “…. Under-representation of Cx. torrentium in carbon dioxide baited traps is commonly observed”).

Authors' response: *Many thanks for pointing this out. We have changed "Alps" to "Swiss Alps" (see line 432).*

Reviewer #2: The manuscript of Muller et al. reports a 6-years survey aimed at recording the potential presence of invasive mosquitoes (Aedes albopictus, Aedes koreicus and Aedes j. japonicus) in Switzerland at selected sites. The study is straightforward and properly conducted. It is evident the huge work done to obtain this set of data, which demonstrated its usefulness in detecting all the three invasive species, allowing also to hypothesize their spreading strategies through the country. I have only a few minor comments:

Lines 93-96: the introduction seems to me unbalanced in describing dispersal and invasiveness of Aedes albopictus while no information is given for the other two Aedes on which the manuscript is focused. I suggest a few more words about distribution and potential invasiveness of Ae. koreicus and Ae. japonicus in order to give a more complete picture of the potential risk of invasion in Switzerland.

Authors' response: *Many thanks for this valid point. We have added two paragraphs in the Introduction section to provide a more balanced picture with regards to all three species (see lines 94 – 115).*

Table 1: please explain in caption the meaning of the asterisk next to the number in BG sentinel column (I guess to highlight the traps with the presence of CO2 supply).

*Authors' response: An explanation has been added in the legend of Table 1: " The star (*) next to the number indicates that the trap was additionally supplied with CO_2_."*

Lines 245-257: it could be interesting to know if the BG sentinel traps baited with BG lure+CO2 collected differently to the others with just the BG lure and if this affected data analysis in terms of detection of invasive species.

Authors' response: *We agree this is an interesting and very relevant question. However, because the traps with and without CO_2_ could not be rotated and, therefore, spatial effects were not accounted for (i.e. balanced out), we feel such a comparison would be inconclusive. On that background, we neither investigated nor report specifically on these differences in the main manuscript. However, on line 447 – 449 we point out that we are aware of the potential issue that CO_2_ could bias the catch rate: "Moreover, the traps had to be well concealed from the public to avoid vandalism. Similarly, only a small subset of BG-Sentinel traps could be fitted with a CO_2_ bottle, meaning that they likely differed in their attraction to mosquitoes. Altogether, this might have introduced a sampling bias." At the same time we deleted the last sentence to have all potential sources of bias together (see track changes). Should a reader have interest in the actual numbers she or he could still look up the raw data in the supplementary material.*

**Additional changes**

The maps in Figures 3-6 accidentally included the Principality of Lichtenstein. We removed Lichtenstein and updated the maps accordingly. The data and the messages remain the same.

There was a mistake in the symbol legend in Figure 5. The smaller red circle should read "10%" and not "1%". The figure has been updated accordingly.

There was a glitch in Table 1 with regards to the altitudes for several sites. We have updated the table with the correct figures. One entry of the altitudes (i.e. " A1 Kölliken-Süd") was also wrong in the supplementary file 1 and we have also updated that table accordingly.

We revised the manuscript for typos and made a few minor stylistic improvements.

All changes are marked using track changes in the respective file.
